# Is strength training more effective than aerobic exercise for improving glycaemic control and body composition in people with normal-weight type 2 diabetes? Reply to Pontes‑Silva A, Santos‑de‑Araujo AD, Teixeira BC et al [letter]

**DOI:** 10.1007/s00125-024-06181-w

**Published:** 2024-06-18

**Authors:** Yukari Kobayashi, Jin Long, Neil M. Johannsen, Ruth Talamoa, Kyla Kent, Cynthia Lamendola, Francois Haddad, Timothy S. Church, Latha Palaniappan

**Affiliations:** 1 Division of Cardiovascular Medicine, Stanford University School of Medicine, Stanford, CA USA; 2 Stanford Cardiovascular Institute, Stanford, CA USA; 3 Department of Pediatrics, Stanford University School of Medicine, Stanford, CA USA; 4 Pennington Biomedical Research Center, Baton Rouge, LA USA; 5 Louisiana State University, Baton Rouge, LA USA; 6 Division of Primary Care and Population Health, Stanford University School of Medicine, Stanford, CA USA; 7 Department of Medicine, Stanford University School of Medicine, Stanford, CA USA; 8 Wondr Health, Dallas, TX USA

**Keywords:** Exercise, HbA_1c_, Normal-weight diabetes

*To the Editor:* We thank Professor Pontes-Silva et al [1] for their letter on our article entitled ‘Strength training is more effective than aerobic exercise for improving glycaemic control and body composition in people with normal-weight type 2 diabetes: a randomised controlled trial’ (STRONG-D trial; ClinicalTrials.gov NCT02448498) [2]. Regarding the first comment by Pontes-Silva et al [1] on the comparison of total work across the three study groups, we agree that exercise interventions are dose-dependent. As mentioned in the methods section [2] and outlined in the STRONG-D methods paper [3], the intensity of the aerobic training was based on participants’ baseline aerobic exercise test, with a target training intensity between 50% and 80% of their peak metabolic equivalents of task (METs). In the aerobic training (AER) group, the total work completed was clamped to 50.2 (12) kJ (kcal) kg (body weight)^–1^ week^–1^, similar to the AER group in the HART-D study [4] conducted in individuals with overweight/obesity and type 2 diabetes. The protocol for the strength training (ST) group [2] was also similar to that in the HART-D study and was never meant to match the amount of work performed in the AER group [4]. We can estimate the total work performed in the ST group as the mean total weight lifted (~40,000 kg/week) × gravity (~10 m/s) × distance travelled (major assumptions are that the eccentric and concentric phases both count as force, and the total distance travelled is 1 m), which is approximately 420 (100) kJ (kcal)/week and 6.0 (1.43) kJ (kcal) kg (body weight)^–1^ week^–1^ (assuming a mean body weight of 70 kg). This suggests that the total work completed by individuals in the ST group (approximately 6.0 (1.43) kJ (kcal) kg^–1^ week^–1^) was considerably less than that in the AER group (approximately 50.2 (12) kJ (kcal) kg^–1^ week^–1^). However, we feel that we do not have enough information to accurately assess this difference or use these data in any adjustments for ‘dose’ in our study. Furthermore, the exercise interventions were also intended to contrast in this study, with the combined strength and aerobic training (COMB) group representing current recommendations for combined strength and aerobic exercise from the American College of Sports Medicine and ADA [5].

Regarding the second comment from Pontes-Silva et al [1] on the statistical analysis used to compare HbA_1c_ reductions across the groups, we understand the limitations of *p* values for determining statistical significance in clinical studies. As Pontes-Silva et al point out [1], there are other ways to evaluate effect sizes in clinical trials than by using *p* values [6]. However, as far as we know, the use of *p* values for determining statistical significance is still the most accepted and understandable method for comparing differences between groups, and *p* values have been previously reported in exercise trials in type 2 diabetes [4]. For the within-group changes in HbA_1c_, 95% CIs (from which SDs can be derived) are reported in Table 2 in our study [2]. For the mean HbA_1c_ comparisons between groups, we used a repeated ANOVA model as detailed in the methods section [2], and the effect size was calculated as the mean difference between groups divided by the SD. Based on the SE estimated using the delta method, the SD for each pairwise comparison can be approximately estimated as (SE)^0.5^. The mean differences in HbA_1c_ between the ST and AER groups are 2.51 (0.23) mmol/mol (%) for the intention-to-treat analysis and 3.06 (0.28) mmol/mol (%) for the per-protocol analysis. The SDs are 3.28 (0.30) mmol/mol (%) for the intention-to-treat analysis and 3.46 (0.32) mmol/mol (%) for the per-protocol analysis. Therefore, for the intention-to-treat analysis, the estimated effect size for the ST vs AER group comparison is 0.77, which is a moderate to large effect size (*p*=0.011), and for the per-protocol analysis, the effect size for the ST vs AER group comparison is 0.88 which is a large effect size (*p*=0.006), as shown in Fig. 2 in our study [2].

Finally, regarding the third comment from Pontes-Silva [1] on the statistical methods used, as stated in the methods section [2], paired *t* tests were used to compare HbA_1c_ levels at baseline and 9 months within groups. Because of the large number of missing data due to study interruption by the COVID-19 pandemic, we used repeated ANOVA for comparisons of mean HbA_1c_ levels over time between groups (Fig. 2) [2]. We understand the concern about type 1 errors raised by Pontes-Silva et al [1]; therefore, as stated in the methods section [2], a Bonferroni-adjusted significance level was used to account for the fact that three comparisons were being made. This is consistent with one of the possible approaches described by CONSORT for use in multi-arm studies [7]. Repeated ANOVA for comparisons of mean HbA_1c_ levels between groups enabled the use of values at all time points, as there were many missing values at 9 months due to the early closure of the study as a result of the COVID-19 pandemic. This is stated in the discussion section: ‘One of the limitations of the STRONG-D study is that it was significantly impacted by the COVID-19 shelter-in-place restrictions introduced in March 2020, which led to early study closure. The follow-up rate was about 45%; therefore, the study was underpowered to obtain conclusive findings’ [2].

## Abbreviations

AER: Aerobic training
ST: Strength training

## Authors’ relationship and activities

The authors declare that there are no relationships or activities that might bias, or be perceived to bias, their work.
